# Cyst of the Canal of Nuck in an Adult Woman: Diagnostic Workup and Surgical Management—A Case Report

**DOI:** 10.1155/cris/8438713

**Published:** 2026-11-15

**Authors:** Evaldo Giacomucci, Sara Tolentino, Francesco Maria Bulletti, Laura Mastrangelo, Maria Cristina Scifo, Chiara Ghizzi

**Affiliations:** ^1^ Gynecology of Infertility Unit Maggiore Hospital Carlo Alberto Pizzardi (Azienda USL di Bologna) Bologna Italy; ^2^ Division of Gynecology and Human Reproduction Pathophysiology IRCCS University Hospital of Bologna Bologna Italy; ^3^ Department of Medical and Surgical Sciences DIMEC University of Bologna Bologna Italy unibo.it; ^4^ Department of Gynecology and Obstetrics University Hospital of Fribourg Fribourg 1708 Switzerland; ^5^ Department of Surgery IRCCS University Hospital of Bologna Sant’Orsola–Malpighi University Hospital Bologna Italy aosp.bo.it; ^6^ Department of Maternal and Child Health Maggiore Hospital Carlo Alberto Pizzardi (Azienda USL di Bologna) Bologna Italy

**Keywords:** canal of Nuck, case report, computed tomography, female inguinal mass, hydrocele, surgery, ultrasound

## Abstract

Cysts of the canal of Nuck are rare congenital abnormalities resulting from incomplete obliteration of the processus vaginalis in females and may mimic more common causes of inguinal swelling, particularly inguinal hernia. We report the case of a 35‐year‐old woman presenting with a symptomatic right inguinal swelling. Ultrasonography demonstrated a well‐defined avascular cystic lesion, and, in this case, contrast‐enhanced computed tomography (CT) was used for further anatomical characterization, showing a thin‐walled fluid‐filled structure extending along the right inguinal canal without bowel herniation or radiological communication with the peritoneal cavity. A cyst of the canal of Nuck was therefore suspected preoperatively. The patient underwent complete surgical excision through an open inguinal approach, without mesh repair. Histopathological examination confirmed a benign mesothelial‐lined cyst consistent with a cyst of the canal of Nuck. At 1‐month follow‐up, the patient was completely asymptomatic, with no clinical or ultrasonographic evidence of recurrence. This case emphasizes the importance of including this uncommon entity in the differential diagnosis of female inguinal masses and illustrates how first‐line ultrasonography, with selective cross‐sectional imaging when additional anatomical definition is required, can support individualized surgical planning.

## 1. Introduction

Cysts of the canal of Nuck represent a rare cause of inguinal swelling in women and constitute the female counterpart of the hydrocele of the spermatic cord in males [1–3]. They result from incomplete obliteration of the processus vaginalis, an embryological peritoneal extension that accompanies the round ligament through the inguinal canal toward the labia majora. The persistence of this structure may lead to fluid accumulation and cyst formation [1, 2].

Because of their rarity and nonspecific clinical presentation, cysts of the canal of Nuck may be mistaken for more common causes of female inguinal masses, particularly inguinal hernia [3–5]. Ultrasonography is generally the first‐line imaging modality, while computed tomography (CT) or magnetic resonance imaging may further characterize the lesion and its anatomical relationships [3, 6, 7]. Surgical excision provides a definitive diagnosis and treatment [3, 8].

We report a case of a cyst of the canal of Nuck in a young woman, focusing on the diagnostic contribution of multimodal imaging, the differential diagnosis of female inguinal masses, and surgical management.

By integrating physical examination with ultrasonography and cross‐sectional imaging, this case illustrates a practical diagnostic pathway for distinguishing a canal of Nuck cyst from more common female groin masses and for tailoring the subsequent surgical approach to the individual anatomy.

## 2. Case Presentation

A 35‐year‐old woman was referred for evaluation of a progressively symptomatic right inguinal swelling present for approximately 3 months. She reported intermittent discomfort and a sensation of local pressure, exacerbated by prolonged standing and physical activity. She denied fever, gastrointestinal symptoms, urinary complaints, or prior similar episodes.

Her medical history was unremarkable. She had no previous abdominal or pelvic surgeries, no known drug allergies, and was not taking regular medications. Menstrual cycles were regular, and obstetric history was noncontributory.

On gynecological examination, the uterus was anteverted and normal in size, with no adnexal tenderness or pelvic masses. Inspection and palpation of the right inguinal region revealed a soft, nonreducible, mildly tender cystic mass measuring ~3 cm in diameter along the course of the inguinal canal. The overlying skin was normal, and the mass did not enlarge during the Valsalva maneuver.

Doppler ultrasonography demonstrated a well‐defined anechoic cystic lesion measuring 30 × 22 × 30 mm^3^ in the right inguinal region without internal septations, solid components, or vascular flow (Figure 1). No pelvic abnormalities were identified. Dynamic ultrasonography during the Valsalva maneuver was not performed.

**Figure 1:**
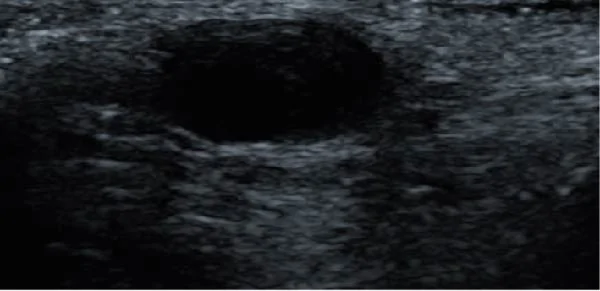
Ultrasound image showing a well‐defined anechoic cystic lesion in the right inguinal region without internal vascularity.

For further anatomical characterization, contrast‐enhanced CT of the abdomen and pelvis was performed. CT demonstrated a thin‐walled fluid‐attenuation lesion measuring 4.0 cm in transverse diameter, 2.2 cm in anteroposterior diameter, and 5.0 cm in craniocaudal extension along the right inguinal canal, without bowel herniation or radiological communication with the peritoneal cavity (Figure 2). CT was selected to define the lesion’s extent and relationship with the inguinal canal for surgical planning; because it provided adequate anatomical characterization, additional magnetic resonance imaging was not considered necessary. Based on the clinical and imaging findings, a cyst of the canal of Nuck was suspected preoperatively.

**Figure 2:**
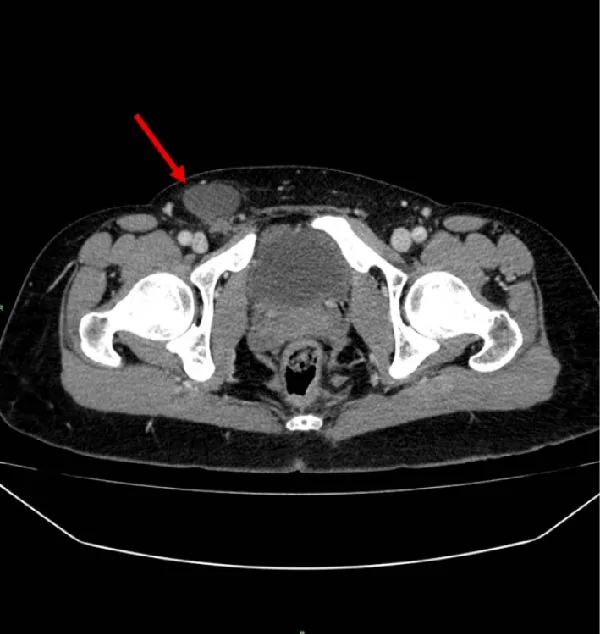
Contrast‐enhanced CT scan demonstrating a thin‐walled fluid‐filled structure along the right inguinal canal, without bowel herniation or peritoneal communication.

Given the patient’s symptoms and the risk of diagnostic uncertainty with conservative management, surgical treatment was recommended.

The operation was performed by the gynaecological team in collaboration with a general surgeon under general anesthesia through an ~2‐cm right inguinal incision. Layered dissection allowed the identification of the internal inguinal ring and an ~4‐cm cyst containing clear citrine fluid (Figure 3).

**Figure 3:**
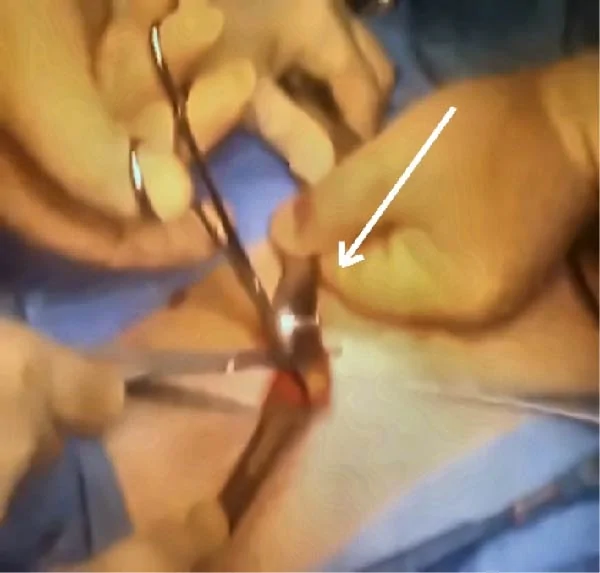
Intraoperative photograph showing the cyst of the canal of Nuck (arrow) within the right inguinal canal.

The cyst was completely enucleated and sent for histopathological examination. The peritoneum and fascia were closed, followed by subcutaneous approximation and absorbable intradermal skin closure. No mesh repair was performed, as no associated inguinal hernia requiring prosthetic repair was identified.

The postoperative course was uneventful, and the patient was discharged on postoperative day one. Histopathological examination of the excised specimen, completed after discharge, demonstrated a benign cystic lesion lined by mesothelial cells, confirming the diagnosis of a cyst of the canal of Nuck (Figure 4). No endometriosis, significant inflammation, hemorrhage, atypia, or malignancy were identified. At a 1‐month follow‐up, clinical examination and ultrasonography showed complete symptom resolution and no evidence of recurrence.

**Figure 4:**
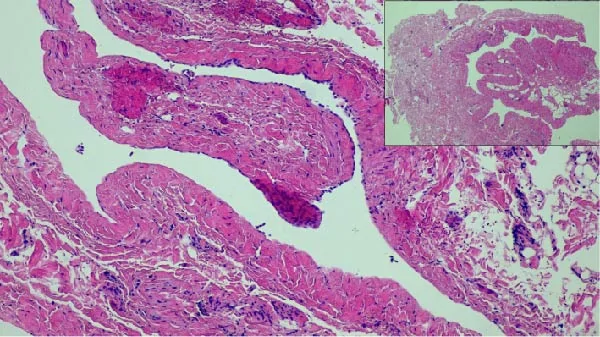
Histopathological section showing a cystic wall lined by mesothelial cells (hematoxylin and eosin stain; original magnification ×100; inset ×40), consistent with a cyst of the canal of Nuck.

The clinical course is summarized in Table 1.

**Table 1:** Clinical timeline.

Time point	Clinical event
Approximately 3 months before evaluation	Onset of right inguinal swelling with intermittent discomfort and local pressure
Initial clinical evaluation	Gynecological and inguinal examination; ~3‐cm nonreducible mildly tender mass; no enlargement during Valsalva maneuver
Ultrasonography	Avascular anechoic cystic lesion measuring 30 × 22 × 30 mm^3^; no solid components or internal septations
Subsequent CT assessment	Thin‐walled fluid‐attenuation lesion measuring 4.0 × 2.2 × 5.0 cm^3^ along the right inguinal canal; no bowel herniation or radiological peritoneal communication
Elective surgery	Open inguinal excision by the gynaecological team in collaboration with a general surgeon; ~4‐cm cyst with clear citrine content was completely enucleated; no mesh repair performed
Postoperative day 1	Uneventful recovery and discharge
After discharge	Definitive histopathological confirmation of a benign mesothelial‐lined cyst
1 month after surgery	Clinical and ultrasonographic follow‐up: patient completely asymptomatic, with no evidence of recurrence

## 3. Discussion

Adult canal of Nuck cysts remain uncommon, but the clinically relevant issue is not rarity alone: their nonspecific presentation overlaps with more common groin lesions and can lead to different diagnostic and operative pathways. Recent adult reports describe painful or painless inguinal or inguinolabial masses, including lesions initially interpreted as lipoma or hernia, and emphasize the value of establishing the anatomy before surgery [9–13]. In the present case, a canal of the Nuck cyst was suspected before surgery, allowing the operative plan to focus on complete excision and assessment of the inguinal canal rather than on empiric hernia repair.

The differential diagnosis of a female groin mass should be approached systematically. An inguinal hernia is favored by reducibility, a cough or Valsalva impulse, or demonstration of bowel or omentum moving through the inguinal canal; a femoral hernia typically lies below the inguinal ligament. Lymph nodes are usually solid and may retain a fatty hilum and vascularity, whereas lipomas show fat echogenicity or fat attenuation on cross‐sectional imaging. Bartholin region cysts are centered near the posterolateral vaginal introitus rather than within the inguinal canal. Round‐ligament endometriosis may present with cyclical pain and a solid or complex lesion, while vascular lesions demonstrate internal Doppler flow. Other soft‐tissue tumors are generally solid and may enhance after contrast administration [4, 5, 12–14]. In our patient, the nonreducible mass did not enlarge during the Valsalva examination, ultrasonography showed a purely cystic avascular lesion, and CT demonstrated its course along the inguinal canal without bowel content, collectively supporting a canal of the Nuck cyst.

Ultrasonography is therefore the preferred first‐line examination because it distinguishes cystic from solid lesions, assesses vascularity, and can be performed dynamically during Valsalva [6, 10, 14]. Dynamic ultrasonography was not performed in the present case, which is a limitation; however, Valsalva was assessed clinically. Cross‐sectional imaging is not mandatory for every patient. CT can provide rapid anatomical delineation of the lesion extent, fluid attenuation, adjacent structures, and possible herniated contents, whereas MRI avoids ionizing radiation and offers superior soft‐tissue characterization, which may be particularly useful for complex lesions or suspected endometriosis [3, 4, 7, 9, 13]. In this adult patient, CT provided sufficient anatomical information for surgical planning, so additional MRI was not considered necessary.

Recent reports illustrate the heterogeneity of adult presentation and management rather than a single mandatory pathway. Nava et al. [9] used ultrasonography, CT, and MRI before open excision of a noncommunicating lesion, with no prosthetic mesh and no recurrence at 6 months. Hattab et al. [10] reported two adult cases diagnosed with ultrasonography, one treated surgically and one managed with observation after the patient declined excision. Rchidi et al. [12] described an adult lesion initially considered a lipoma before ultrasonography suggested the correct diagnosis, whereas Hang et al. [11] reported different anatomical types, including cases associated with hernia, requiring different operative strategies. Ruiz et al. [13] likewise reported successful open anterior excision in an adult woman. Together, these contemporary reports support tailoring both imaging and treatment to the lesion anatomy, symptoms, and the presence of an associated hernia.

For symptomatic lesions, complete surgical excision remains the most frequently reported definitive treatment [9–13]. An open inguinal approach provides direct access to the cyst and inguinal canal and may be particularly practical for distal or noncommunicating lesions. Laparoscopic approaches can be useful when a communicating or proximal component, intra‐abdominal extension, or concomitant hernia is suspected [11, 15]. Mesh placement should not be considered an intrinsic component of the canal of Nuck cyst excision; rather, hernia repair and the use of mesh should be individualized according to the presence and characteristics of an associated defect. In the present case, no associated inguinal hernia requiring prosthetic repair was identified, and no mesh was used. Although short‐term observation has been reported in a selected patient who declined surgery, the evidence supporting conservative management remains limited [10].

Histopathological examination remains important because it confirms the mesothelial‐lined cyst and can exclude alternative pathological processes. In our specimen, no endometriosis, significant inflammation, hemorrhage, atypia, or malignancy were identified. The patient was completely asymptomatic at 1‐month clinical and ultrasonographic follow‐up, with no evidence of recurrence. Recent adult reports also describe favorable short‐term outcomes after complete excision, although the available literature consists predominantly of individual cases and small series and does not define an evidence‐based duration of follow‐up [9, 10, 13].

This report has several limitations. It describes a single uncomplicated case, postoperative follow‐up was limited to 1 month, dynamic ultrasonography during Valsalva was not performed, and MRI was not required after CT provided adequate anatomical characterization. Consequently, this case cannot establish the superiority of one imaging strategy or surgical approach for all patients with canal of Nuck cysts.

Taken together, this case supports a practical anatomy‐based approach to female inguinal masses: consider a canal of Nuck cyst in the differential diagnosis, use ultrasonography as first‐line imaging with selective cross‐sectional imaging when further anatomical definition is needed, and tailor the operative approach and need for mesh repair to the individual lesion and any associated hernia.

## Author Contributions

**Evaldo Giacomucci and Sara Tolentino**: writing – original draft. **Sara Tolentino, Francesco Maria Bulletti, and Chiara Ghizzi**: investigation, writing – review and editing. **Laura Mastrangelo and Maria Cristina Scifo**: writing – review and editing.

## Funding

This research did not receive any specific grant from funding agencies in the public, commercial, or not‐for‐profit sectors. No funding sources were involved in the study.

## Disclosure

All authors read and approved the final manuscript.

## Ethics Statement

Ethical approval was not required for this case report in accordance with the institutional policies.

## Consent

Written informed consent for the publication of this case report and any accompanying images was obtained from the patient. Written informed consent was obtained from the patient.

## Conflicts of Interest

The authors declare no conflicts of interest.

## Data Availability

All data relevant to this case report are included in the article. Additional clinical information is not publicly available to protect patient privacy, but de‐identified data may be provided by the corresponding author upon reasonable request.
